# Dual labor market and the “Phillips curve puzzle”: the Japanese experience

**DOI:** 10.1007/s00191-022-00781-8

**Published:** 2022-08-05

**Authors:** Hideaki Aoyama, Corrado Di Guilmi, Yoshi Fujiwara, Hiroshi Yoshikawa

**Affiliations:** 1 RIKEN, Interdisciplinary Theoretical and Mathematical Sciences Program (iTHEMS), Saitama 351-0198 Wako, Japan; 2 Research Institute of Economy, Trade and Industry (RIETI), 100-0013 Tokyo, Japan; 3 Economics Discipline Group, University of Technology Sydney, Broadway, NSW 2007 Australia; 4 Centre for Applied Macroeconomic Analysis, Australian National University, Canberra, Australia; 5 Center for Computational Social Science, Kobe University, 657-8501 Kobe, Japan; 6 Graduate School of Information Science, University of Hyogo, 650-0047 Kobe, Japan; 7 The University of Tokyo, 113-0033 Tokyo, Japan

**Keywords:** Phillips curve, Bargaining power, Secondary workers, C60, E31

## Abstract

Low inflation was once welcomed by both policymakers and the public. However, Japan’s experience during the 1990s changed the consensus of economists and central banks around the world regarding prices. Facing deflation and the zero-interest bound at the same time, the Bank of Japan had difficulty conducting an effective monetary policy, making Japan’s stagnation unusually prolonged. The too-low inflation that concerns central banks today translates into the “Phillips curve puzzle.” In the United States and Japan, in the course of the recovery from the Great Recession after the 2008 Global Financial Crisis, the unemployment rate had steadily declined to a level commonly regarded as lower than the natural rate or NAIRU. However, inflation remained low. In this paper, we consider a minimal model of the dual labor market to jointly investigate how the different factors affecting the structural evolution of the labor market have contributed to the observed flattening of the Phillips curve. We find that the level of bargaining power of workers, elasticity of the supply of labor to wage in the secondary market, and composition of the workforce are the main factors jointly explaining the evidence for Japan.

**Supplementary Information:**

The online version contains supplementary material available at 10.1007/s00191-022-00781-8

## Introduction

Low inflation was once welcomed by both policymakers and the public. However, Japan’s experience during the 1990s changed the consensus of economists and central banks around the world. After the financial bubble burst at the beginning of the 1990s, Japan experienced deflation, during which, the Bank of Japan (BOJ) continued to cut the nominal interest rate down to zero. Facing deflation and the zero-interest bound at the same time, the BOJ found it difficult to effectively conduct monetary policy, making Japan’s stagnation unusually prolonged.

The “Japan problem” made economists aware of the long-forgotten danger of deflation. In the prewar period, deflation was a menace to the economy, and its danger was emphasized by famous economists such as Keynes (1931) and Fisher (1933). To prevent deflation, central banks must seek low inflation rather than zero inflation or a stable price level. Following this idea, many central banks, including the U.S. Federal Reserve, BOJ, and European Central Bank, target 2% inflation of the consumer price index. However, few central banks have achieved this goal satisfactorily.

The too-low inflation that concerns central banks today translates into the “Phillips curve puzzle”. The benchmark Phillips curve is expressed as (Phillips 1958; Frıedman 1968):

1 $$\pi_{t}=-a (u-u^{*})+b \pi_{t}^{*},$$

where *π* and *u* are the inflation and unemployment rates, respectively. *π*^∗^ is either the inflationary expectation or inertia of past inflation. *u*^∗^ is the natural rate of unemployment or non-accelerating inflation rate of unemployment (NAIRU), and $a,b>0$. According to conventional macroeconomics, Eq. (1) or the Phillips curve plays an important role in determining inflation.

In the U.S. and Japan, in the course of recovery from the Great Recession after the 2008 Global Financial Crisis, the unemployment rate steadily declined to a level commonly regarded as lower than the NAIRU, *u*^∗^. However, inflation remained low at 0.5% for Japan and 1.5% for the U.S.

**Fig. 1:**
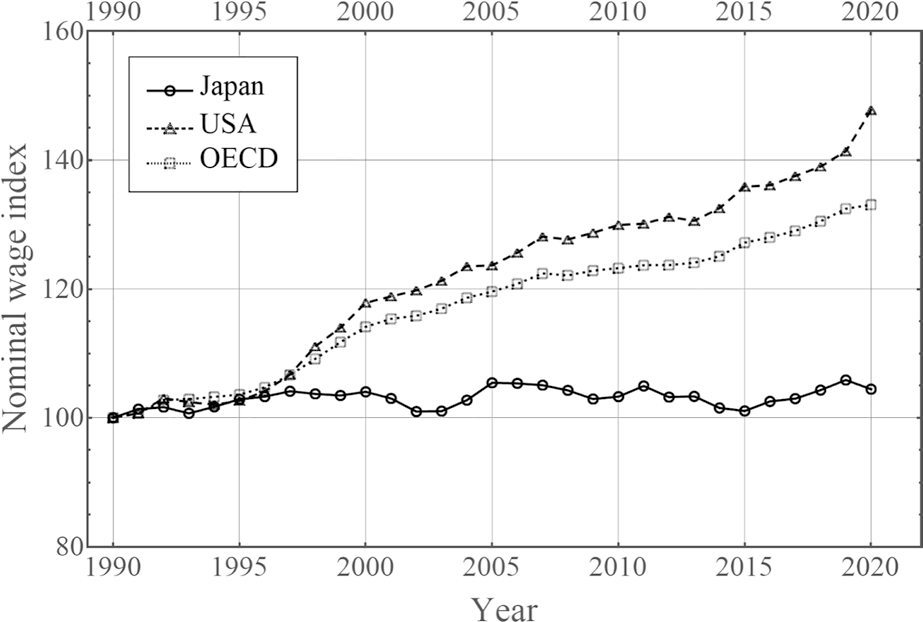
Nominal wage index in Japan,the U.S., and the Organization for Economic Co-operation and Development (OECD) (1990 = 100). Source: OECD

The Phillips curve puzzle is acute, particularly in Japan, because the core of Japan’s notorious deflation is the stagnation of nominal wages. For example, in 2019, the U.S. PCE of manufactured products declined by 0.5%, whereas its Japanese counterpart rose by 1.0%. However the consumer price of labor-intensive services rose by 2.4% in the U.S., whereas it rose only by 0.5% in Japan. This difference reflects the stagnation of nominal wages in Japan. Figure 1 shows that Japan is unique among advanced economies in that nominal wages have stagnated, and occasionally even declined, for a prolonged period. Therefore, to understand Japan’s deflation, we must analyze nominal wages. In contrast, for the U.S., Coibion and Gorodnichenko (2015), Del Negro et al. (2020), and Hooper et al. (2020) focus on the price Phillips curve and regard the flattening of the wage Phillips curve as irrelevant. As Fig. 1 demonstrates, cross-country differences exist and may contribute explaining the deflation. In this study, we analyze the wage Phillips curve rather than the price Phillips curve.

In terms of the Phillips curve (Eq. 1), two patterns have been detected. First, the coefficient *b* for inflationary expectations or lagged inflation has declined significantly almost to almost zero in recent years. Blanchard (2018 Figure 7), for example, finds that *b*, which was almost zero in the early 1960’s, rose sharply to one in the late 1960s, stayed that way for 30 years, and then declined suddenly to zero at the beginning the 2000s. While the decline in inflation since the 1990s has been often attributed to better policy management —even the term “Great Moderation” was coined (Clarida et al. 2000)—more recent analyses have identified the anchoring of inflation expectations as one of the main factors for the change in the trade-off between inflation and unemployment (Barnichon and Mesters 2021; Blanchard 2016). Greenspan (2001) remarks: “Price stability is best thought of as an environment in which inflation is so low and stable over time that it does not materially enter into the decisions of households and firms.”

The second factor is the change in coefficient *a* for the unemployment rate in Eq. (1). A decline in *a* entails lower inflation than otherwise when the unemployment rate declines. Some researchers even argue that unemployment no longer affects inflation, at least within some unemployment and inflation ranges.

**Fig. 2:**
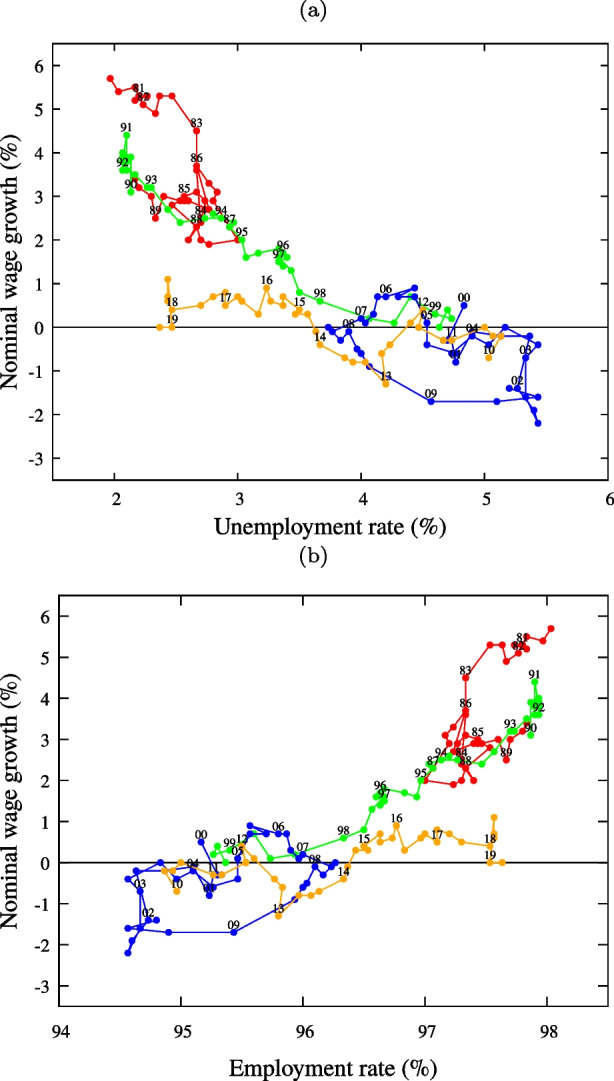
**Japan’s wage Phillips curve: Unemployment rate and nominal wage growth of 157 quarters from 1980.π to 2019.π.** (a) The abscissa represents the unemployment rate in %. (b) The abscissa is reversed and represents the employment rate (= 100-unemployment rate (%)). Colors: 1980s (red), 1990s (green), 2000fs (blue), 2010fs (orange). The last several years’ data (in yellow) show definite deviation from those of the earlier years, with low nominal wage growth despite of the low unemployment. Sources: (Ministry of Health Labor and Welfare 2020) and (Statistics Bureau of 2021)

Figure 2 (a) displays Japan’s wage Phillips curve, namely, the quarterly relationship between the unemployment rate and nominal wage growth for 1980.II–2019.II. We can indeed observe that the wage Phillips curve has flattened in recent years. As we analyze employment rather than the unemployment rate in the model, for the reader’s convenience we also show the Phillips curve in terms of employment in Fig. 2 (b).

These issues define the “Phillips curve puzzle.” In this paper, we leave the first problem untouched; we simply take *b* as zero. Figure 1 demonstrates that in the case of Japan, the *expected* wage inflation is a minor factor, simply because the level of nominal wages has stagnated for two decades, The BOJ’s new monetary policy, introduced in April 2013 under the new governorship of Mr. Haruhiko Kuroda, aimed to raise price/wage hike expectations by doubling the monetary base, but failed to deliver for more than eight years. Thus, ignoring *b*, we focus on the second problem, namely, why *a* becomes smaller in Eq. (1).

For this purpose, we consider a minimal model of the labor market. We believe that it is useful and important to establish a minimal model to organize various issues concerning the Phillips curve puzzle. In a simple model of the dual labor market (as in McDonald and Solow 1981, 1985; Gordon 2017), we explore the kinds of changes in the economy that flatten the Phillips curve. The analysis of the model reveals that the level of bargaining power of workers, the elasticity of the supply of labor to wages in the secondary market, and the composition of the workforce are the main factors explaining the Philips curve.

Our main contribution is to provide a compact model that considers the joint evolution of structural factors that have been hitherto investigated separately in the literature.^1^ The main finding is that the change in the shape of the relationship between the level of economic activity and wage inflation can only be explained by the joint effects of these four factors. More specifically, none of these factors by themselves appear to generate the shift in the wage Phillips curve observed in the data. This conclusion introduces a new and original perspective because, to the best of our knowledge, the theoretical literature has so far either focused on subsets of factors at the time, or provided a microeconomic explanation based on changes in the behavior of employers and workers. Our study suggests that any attempt to explain the dramatic shift in the relationship between wage inflation and the level of economic activity observed for Japan should focus on the deep structural modifications in the labor market that have occurred over the last three decades.

The remainder of this paper is organized as follows. Section 2 introduces the theoretical model and the analytical solution. Section 3 presents some comparative statics and sensitivity analysis, which are discussed in Section 4. Finally, Section 5 provides some concluding remarks.

## The model

The model assumes a dual labor market consisting of primary labor and secondary workers (McDonald and Solow 1981, 1985; Gordon 2017). As in Di Guilmi and Fujiwara (2022), we identify as secondary workers all the employees without a permanent contract (agency, temporary, and part-time workers).^2^

### Model structure

Firms are heterogeneous in size and efficiency, but adopt the same production function. Each firm produces a homogeneous good by employing only labor, composed of primary and secondary workers. Specifically, firm *j*(*j* = 1,⋯ ,*N*) employs *L*_1,*j*_ primary workers at wage *w*_1,*j*_ and *L*_2,*j*_ secondary workers at wage *w*_2_. There are $L_1={\sum }_{j=1}^{N} L_{1,j}$ primary workers and $L_2={\sum }_{j=1}^{N} L_{2,j}$ secondary workers employed.

The endowment of primary workers is fixed for each firm.^3^ Accordingly, *L*_1,*j*_ is a given constant. In contrast, firms freely change the level of secondary workers. Following the empirical findings of Munakata and Higashi (2016), the wage of secondary workers is determined by the market and is uniform across firms.

The output of firm *j* is determined as follows:

2 $$Y_{j}=A_{j} (L_{1,j}+c L_{2,j})^{\alpha},$$

where *α* ∈ (0,1). *A*_*j*_ is firm-specific total factor productivity (TFP). Parameter *c* ∈ (0,1) quantifies the productivity of secondary labor relative to that of primary workers. In order to focus on nominal wages, we abstract from price determination in the analysis. Thus, price is implicitly incorporated into output *Y* in Eq. 2, which is in value terms. Firms face different market conditions, and thus, their markups are different. We assume that output prices are reflected in TFP: *A*_*j*_ represents not only the firm’s technology but also its market power.

The profit is given by

3 $${\Pi}_{j} = Y_{j}-L_{1,j} w_{1,j}-L_{2,j} w_2.$$

In order to mimic the firm-level bargaining process prevailing in Japan, primary workers’ wage is set in a two-step process for each employer. Assuming a fixed endowment of primary workers (or *insiders*) *L*_1,*j*_ for each firm, in the first stage firm and primary workers determine the number of secondary workers (or *outsiders*) *L*_2,*j*_ to be hired. Assuming a perfectly competitive market for secondary workers, firms take the secondary wage *w*_2_ as given. Once the number of secondary workers is determined, firms and insiders share the surplus defined by revenue less the wages paid to secondary workers through a Nash bargaining (Mortensen and Pissarides 1994).

#### First-stage maximization

Each firm maximizes its profit (3) by choosing the number of secondary workers or outsiders:

4 $$\max_{L_{2,j}} \left[ {\Pi}_{j} \right].$$

This determines demand for secondary workers $L_{2,j}^{\text {(d)}}$ of firm *j* as follows:

5 $$L_{2,j}^{\text{(d)}}=\frac{1}{c}\left[ -L_{1,j}+\left(\frac{\alpha c A_{j}}w_2\right)^{1/(1-\alpha)} \right].$$

The total demand for secondary workers in the economy as a whole is then:

6 $$L_2^{\text{(d)}} =\frac{1}{c} \left[-L_1+ \bigg(\frac{\alpha c A}w_2\bigg)^{1/(1-\alpha)} \right], \text{with} \quad A=\left(\sum\limits_{j=1}^{N} A_{j}^{1/(1-\alpha)}\right)^{(1-\alpha)}.$$

We assume the following supply function of secondary workers *L*_2_:

7 $$L_2^{\text{(s)}}=B w_2^{\beta}.$$

where *β* is the Frisch elasticity and *B* stands for factors such as demography that affect the supply of secondary workers independent of wages. Matching the demand for and supply of secondary workers, $L_2^{\text {(s)}}=L_2^{\text {(d)}}$, we obtain the following nonlinear equation for *w*_2_:

8 $$B w_2^{\beta}=\frac{1}{c} \left[-L_1+ \bigg(\frac{\alpha c A}w_2\bigg)^{1/(1-\alpha)} \right],$$

**Fig. 3:**
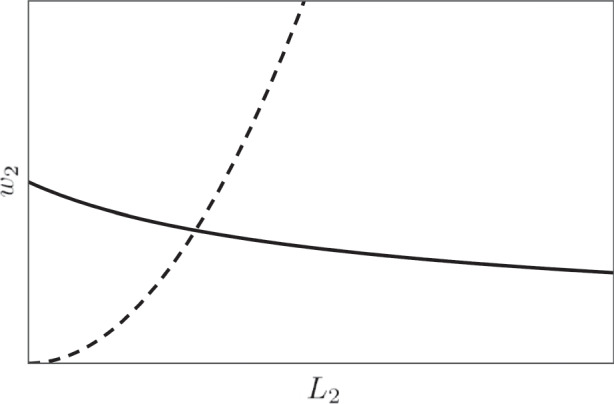
Demand function $L_2^{\text {(d)}}$ defined by Eq. (6) (solid curve) and supply function $L_2^{\text {(s)}}$ of Eq. (7) (dashed curve)

The demand for and supply of secondary labor as functions of *w*_2_ are shown on (*L*_2_,*w*_2_) plane in Fig. 3. It can be observed that the solution of Eq. (8) always exists.

#### Second stage maximization: primary workers’ wage

Firms and primary workers (insiders) determine the wage of primary workers *w*_1,*j*_ through Nash bargaining:

9 $$\max_{w_{1,j}} \left[(L_{1,j} w_{1,j})^{\gamma} ({\Pi}_{j})^{1-\gamma}\right],$$

where *γ* ∈ (0,1) indicates the bargaining power of primary workers.

While spillovers from temporary workers’ contracts to primary workers’ wages have been detected in Europe (see Bellani and Bosio 2019), independence between the two markets is a suitable assumption for Japan. The knock-on effect appears to be relevant only in countries with relatively low job security and Japan typically outperforms European countries according to the general OECD job-security indexes. Furthermore, in Japan, the two markets are at least partially disconnected on the supply side since a large proportion of secondary workers in Japan are seniors and women with home care duties, who are unlikely to compete fror primary as found by Bank of Japan (2018) and further discussed in Section 4.

Nash bargaining determines *w*_1,*j*_ as follows:

10 $$w_{1,j}=\gamma\frac{ Y_{j} -L_{2,j} w_2}{L_{1,j}}.$$

By combining Eqs. (3) and (10), we find that

11 $${\Pi}_{j}=\frac{1-\gamma}{\gamma}L_{1,j}w_{1,j}.$$

We study the relationship between the total employment of workers, *L* = *L*_1_ + *L*_2_ and the average wage $\bar {w}={W}/{L}$, where *W* is the total earnings of all the workers,as follows:

12 $$W=\sum\limits_{j=1}^{N}\left(L_{1,j} w_{1,j}\right)+L_2w_2$$

By combining Eqs. (8) to (12), we obtain the folllowing:

13 $$W=\gamma A \left(\frac{\alpha cA}{w_2}\right)^{\alpha/(1-\alpha)} +(1-\gamma)Bw_2^{1+\beta}.$$

Since *L* and $\bar {w}$ are both functionally dependent on *A*, we can easily determine the functional relationship between *L* and $\bar {w}$. Curve *w*(*L*) is the wage Phillips curve that models the relationship shown in Fig. 2 (b). The wage Phillips curve is expressed in terms of the wage level (rather than wage inflation) because, as shown below, the rate of change in wages is implied by the wage level.

Table 1 lists the parameters and variables of the model. The model is extremely parsimonious with only seven free parameters: *A*,*L*_1_,*c*,*α*,*B*,*β*,*γ*. However, because of nonlinearities, its solution is not trivial and can generate a set of interesting results.

**Table 1:** List of the parameters and variables of the model

Output	
*A*	Nonlinear sum of total factor productivity *A*_*j*_ (Eq. 6)
*L*_1_	Total number of primary workers
*L*_2_	Total number of secondary workers employed (Eq. 5)
*c*	Secondary workers’ productivity coefficient
*α*	Output exponent
Supply of secondary workers	
*B*	Coefficient of labor supply for secondary workers
*β*	Wage elasticity for secondary workers
Nash Bargaining	
*w*_1,*j*_	Wage of the primary workers at firm *j* (Eq. 10)
*w*_2_	Wages of the secondary workers (Eq. 8)
*γ*	Bargaining power of the primary workers

The variables are determined by the equation referred in parentheses

### Solution of the model

To solve the model, we first rewrite Eq. (8) as follows:

14 $$\frac{w_2}{\alpha c A}=\left(L_1 + c B w_2^{\beta}\right)^{-(1-\alpha)}.$$

This equation can be expressed in a simple form:

15 $$v=\left(1+g v\right)^{-\beta(1-\alpha)},$$

where we used the following scaled variables

16 $$\begin{array}{@{}rcl@{}} v\equiv\frac{\ L_1^{\beta(1-\alpha)}}{\ (\alpha c A)^{\beta}\ }w_2^{\beta}, \quad g\equiv c B\left(\alpha c A\right)^{\beta} L_1^{-1-\beta(1-\alpha)}. \end{array}$$

The perturbative solution of (16) is as follows:

17 $$v=1-\sigma g +\frac12 \sigma(1+3\sigma)g^{2} +\cdots,$$

where *σ* ≡ *β*(1 − *α*).

This leads to the following: 4

18 $$\bar{w}=\frac{\gamma}{\alpha c}\left(\frac{L_{1}}{B}\right)^{1/\beta}\left(\frac{L}{L_1}-1\right)^{1/\beta} \left[1 +\frac{c(\alpha +\gamma-\alpha\gamma)-\gamma}{\gamma} \left(\frac{L}{L_{1}}-1\right) +{\cdots} \right].$$

Thus, the wage Phillips curve has the expected sign of slope: it is upward sloping on the employment–wage plane, therefore, is downward sloping on the wage–unemployment plane. We find from the leading term of this expression that the slope of the curve depends on two key factors: *γ*/(*cαB*^1/*β*^) and 1/*β*.

## Comparative Statics

**Fig. 4:**
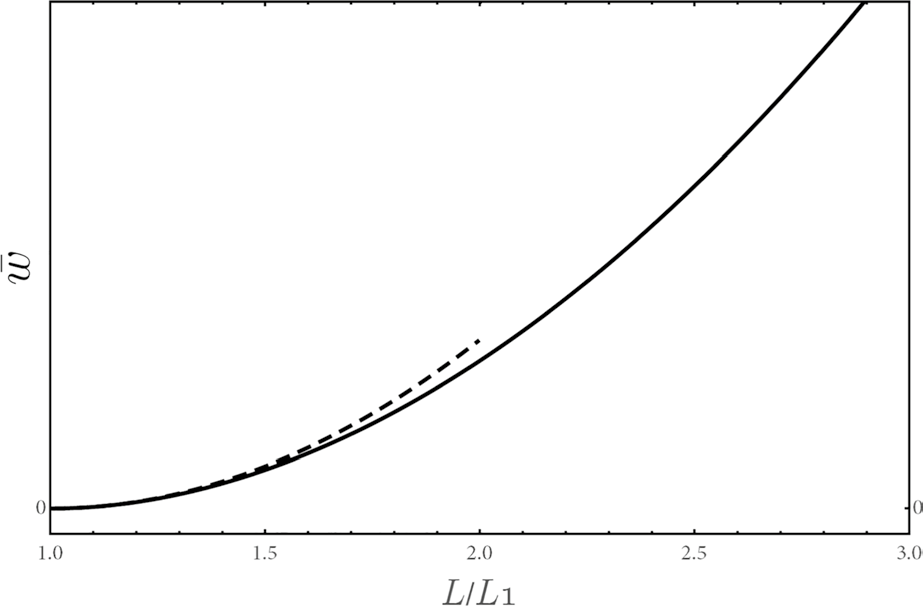
**Numerically simulated wage Phillips curve and analytical approximation.** Solid curve: numerical solution $\bar {w}(L)$; dashed curve: analytical solution (the leading term of Eq. (18)). Parameters are set as follows: *α* = *c* = *β* = *γ* = 0.5

In order to estimate the effects of the parameters, we provide here numerical comparative statics exercise. The estimated parameters are as follows: *c* = 0.5 (following Di Guilmi and Fujiwara 2022), *γ* = 0.5 (Carluccio and Bas 2015); *β* ∈ [0.7,0.1] (Kuroda and Yamamoto 2007), while other parameters are calibrated. The wage Phillips curve is plotted in Fig. 4, where the solid curve is the numerical solution and the dashed curve is the analytical solution Eq. (18). We find that the analytical solution (18) provides a reliable approximation for it mimics well the original function in *L*/*L*_1_ ∈ [1,2].

**Fig. 5:**
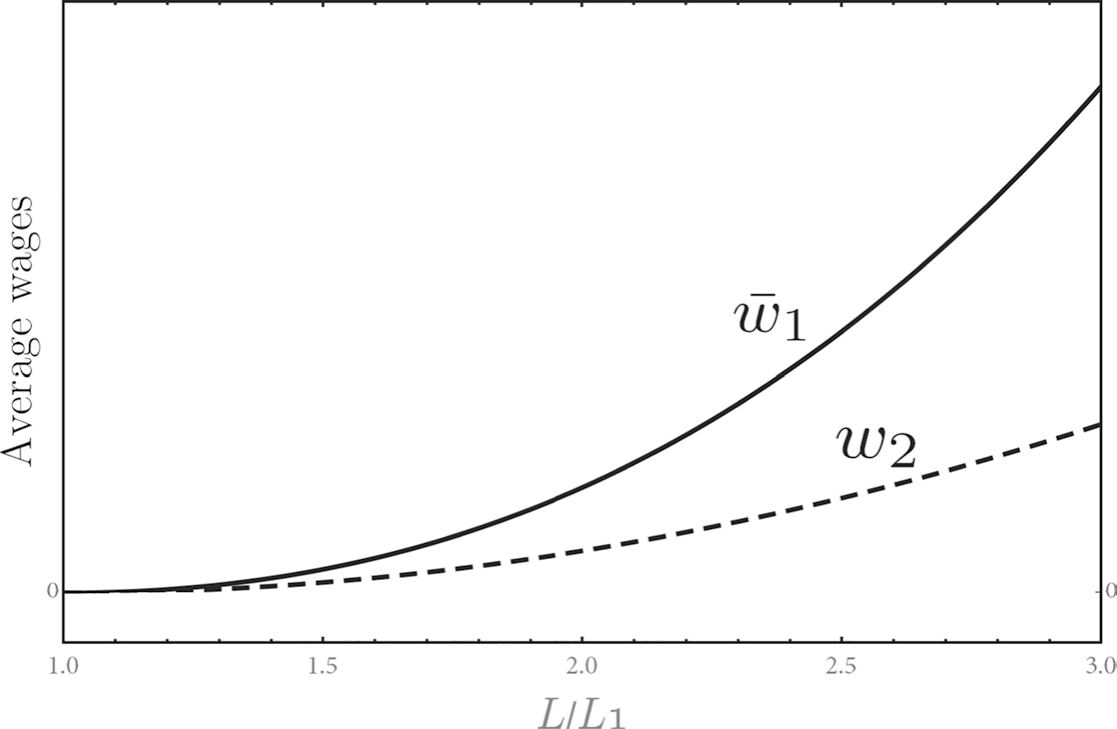
The behavior of the wages $\bar {w}_{1}$ (**solid curve**) and *w*_2_ (**dashed curve**). The parameters are the same as in Fig. 4

The average wage of primary workers,

19 $$\bar{w}_{1}\equiv \frac1{L_{1}}\sum\limits_{j=1}^{N} L_{1,j} w_{1,j}$$

and secondary workers’ wage *w*_2_ are shown in Fig. 5 as functions of total employment *L*. It is interesting to observe that the wages of primary workers determined by Nash bargaining increase more than market-determined wages of secondary workers when total employment increases.

Our goal is to explain why the wage Phillips curve has flattened in recent years. For this purpose, we explore how the slope of the wage Phillips curve depends on the parameters *c*,*B*,*γ*, and *β*.

**Fig. 6:**
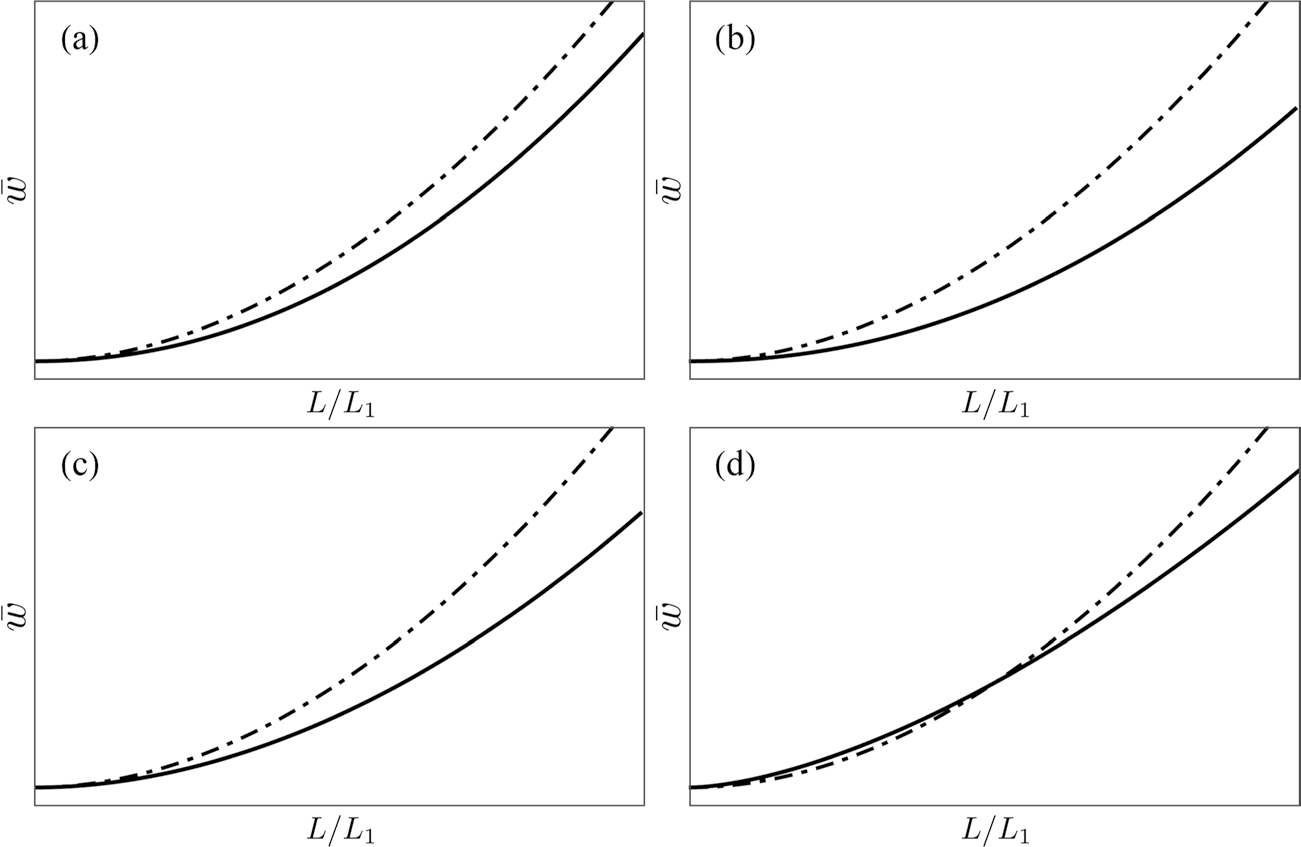
**The parameter dependence of the wage Phillips curve.** The dash-dotted curve is the same as the solid curve Fig. 4, with *α* = *c* = *γ* = *β* = 0.5. The numerical value of *B* is irrelevant to this figure, as we do not specify the scale of the vertical axis, and we just indicate it as *B* = *B*_1_. The solid lines in each panel are obtained by changing the value of one parameter as follows: (a) *c* is increased from 0.5 to 0.9; (b) *γ* is decreased from 0.5 to 0.2; (c) *B* is increased by 20%; (d) *β* is increased from 0.5 to 0.6.

Now, the Philips curve with relatively small changes of the parameters *c*,*γ*,*B* and *β* in the manner explained above are illustrated in Fig. 6 in comparison with the wage Phillips curve in Fig. 4.

**Fig. 7:**
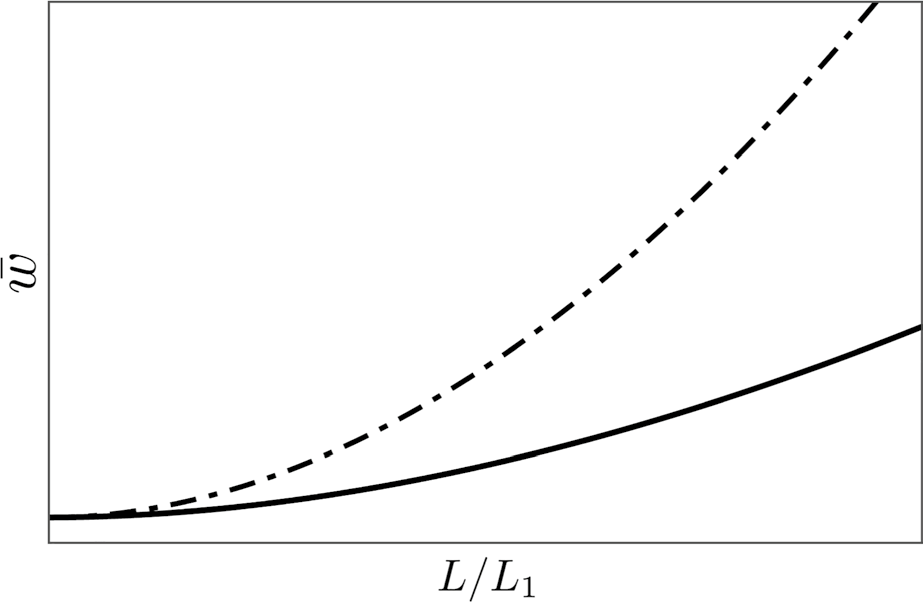
**Combined effect of parameter changes.** The parameters are *α* = *c* = *β* = *γ* = 0.5 for the dot-dashed curve and *α* = 0.5, *c* = 0.9, *β* = 0.6, *γ* = 0.2 with 20% increase in *B* for the solid curve. The combined effect of small parameter changes accumulates and flattens the wage Phillips curve

Combining the effects of the changes in the four parameters *c*,*γ*,*B*, and *β* shown in Fig. 6, we obtain Fig. 7, in which we can clearly observe the flattening of the wage Phillips curve.

## Discussion

The changes in the model model parameters that flatten the wage Phillips curve are related to the structural evolution of the Japanese economy. They are: (1) an increase in the productivity of secondary workers relative to that of primary workers, (2) weaker bargaining power of primary workers, (3) an increase in the supply of secondary workers, and (4) an increase in the wage elasticity of supply of secondary workers. These changes have occurred in the Japanese economy in the last 30 years.

**Fig. 8:**
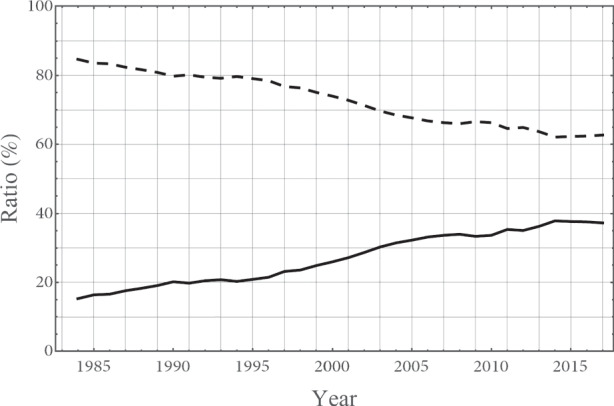
**The ratio of primary workers (solid curve) and secondary workers (dashed curve) to the total number of workers.** Secondary workers include: part-time workers, dispatched workers from labor agency, contract/entrusted employees, and generic ”other” category (Statistics Bureau of 2021)

As shown in Fig. 8, the share of secondary or irregular workers in Japan, which was 15%–16% during the late 1980s, has steadily increased since then to almost 40% in 2020 (see also Kawaguchi and Ueno 2013; Gordon 2017). After the bubble burst at the beginning of the 1990s, Japanese firms facing unprecedented difficulties attempted to cut labor costs by replacing highly paid primary workers with low-wage secondary workers. Historically, secondary workers have been considered to have lower productivity because of less education/training and lower attachment to the employer (Fukao and Ug Kwon 2006; Shinada 2011). However, in the face of a dramatic increase in the secondary workforce, overall productivity has been mostly stagnant and not reflected the decline in the proportion of primary workers. This is consistent with an improvement in the relative productivity of secondary workers. The entry of educated women into the workforce and the re-entry of skilled older workers could have contributed to the higher average productivity of secondary workers. As these categories of workers had previously been out of the workforce, the change in relative productivity could occur without affecting the primary market.

The increase in the secondary employment and workforce also has relevant implications for wage setting. Chen (2018) finds that secondary workers typically have low bargaining power, which employers exploit to suppress wage growth. In contrast to the conclusions of Hirata et al. (2020) and Iwasaki et al. (2021), who emphasize downward wage rigidity, Chen (2018) attributes upward wage rigidity to an increase in the proportion of secondary workers with lower wage growth compared to permanent workers.

Over the last few decades, the bargaining power of primary workers has also been affected by the general de-unionization of capitalist economies.^5^ In the case of Japan, unions have faced a higher pressure to restrain wage demand compared to Western economies in order to limit the loss of jobs following the double burst of the stock and real estate market bubbles at the onset of The Lost Decade.

As for the labor supply and wage elasticity of secondary workers, the Bank of Japan (2018) report of July 2018 on wages and prices provides empirical support for our theoretical findings. Bank of Japan (2018) explicitly points to the dual labor market with different wage-setting mechanisms as the reason for the sluggish wage dynamics. In particular, the report argues that the increase in labor force participation rates among seniors and women is the main cause underlying the weaker reaction of wages to employment. Indeed, in the last two decades, post-war baby boomers have reached the age of retirement, left primary jobs, and entered the secondary economy. At the same time, in line with other developed economies, an increase in women’s participation has created additional workforce availability, particularly for part-time employment. In addition to the increase in the number of retired post-war baby boomers and women, a substantial number of prime-age men who would once have been primary workers have joined the secondary workforce, because Japanese firms facing severe difficulties tried hard to cut labor costs after the bubbles burst and substituted primary workers with low-wage secondary workers.

Consequently, the supply of secondary workers has increased. The Bank of Japan (2018) estimates a relatively higher wage elasticity for secondary labor, such as seniors and women, than male workers in working age. Therefore, an increase in labor demand does not translate into a high wage increase. The relatively large wage elasticity of the supply of older adult workers has also been confirmed in other OECD countries (Mojon and Ragot 2019).

In order to compare the results obtained in the model with the evolution of the four factors discussed above, we focus on the first term in Eq. (18). We consider the period 2018–2019 (let us denote this time-window as “period II”) when the unemployment rate was about 2.5% and the nominal wage growth was around 0.5%. The unemployment rate was approximately 2.5% in the late 1980s (in red in the lower panel of Fig. 2) to the early 1990s (in green in the lower panel of Fig. 2, identified as “period I”) but the nominal wage growth was around 3%. Given a 2.5% unemployment rate, wage growth was 2.5 % lower in period II than in period I: The Phillips curve had flattened. We explore how this change is generated in our model.

For this discussion, we denote the values of the parameters in each period using the subscripts I and II; for example, the *c* in Periods I and II are denoted by *c*_*I*_ and *c*_*II*_, respectively. Let us assume that the average wage for one period is given and take one year as the time reference. In period I, it increases by 3% the next year. In Period II, it increases only by 0.5%. Thus, the next year’s wage in Period II is $1.005/1.03\simeq 0.976$ times that of the next year in period I. We explore how this difference is explained by simultaneous changes in *c*,*γ*, and *B*, keeping *β* fixed in order to focus on possible structural changes in the labor market. By taking the first term in Eq. (18) we find that

20 $$\frac{\mathrm{I}{\text{ratio}}}{c_{\text{ratio}} B_{\text{ratio}}^{1/\beta}}=\frac{1.005}{1.03}=0.976.$$

where we denote the ratios of the parameters between the two periods *c*_ratio_ = *c*_II_/*c*_I_, *B*_ratio _= III/II,*B*_ratio_ = *B*_II_/*B*_I_.

**Fig. 9:**
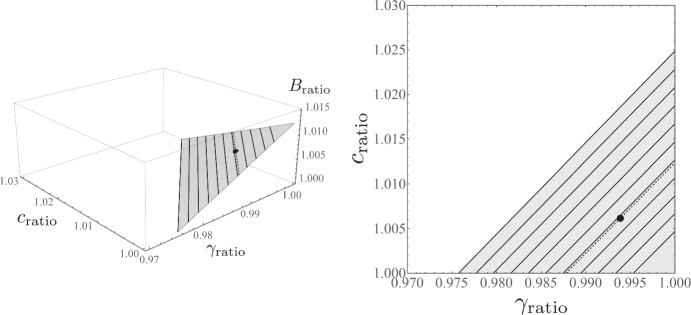
Changes in parameters Iratio,*c*_ratio_, and *B*_ratio_ that explain the flattening of the wage Phillips curve between Periods I and II, given Eq. (20). The left-hand panel shows the surface that the solution covers, with the solid lines for *B*_ratio_ = 1,1.001,1.002⋯ ,1.01, a black sphere for the case in which *c*,*B*, and *γ* are changed simultaneously at a similar level as explained in the text (see Eq. (20)), and the dashed line for the required value of change in *B* (*B*_ratio_ = 1.00616). The right-hand panel is the projection onto the (*γ*_ratio_,*c*_ratio_)-plane

Suppose that the three parameters change in the same ratio *c*_ratio_ = *B*_ratio_ = 1/Iratio = *r* with *β* = 0.5. From (20), we obtain *r* = 1.00616, that is, a 0.62% increase in *c* and *B* with a 0.61% reduction in *γ*. From this, we learn that even though the change in each parameter is small, it reduces the wage growth significantly from 3% to 0.5%. The general solutions to Eq. (20) are shown in Fig. 9. This computational experiment suggests that the changes in the Japanese wage Phillips curve should be understood as a combined effect of relatively marginal changes in the labor market.

To summarize, our findings emphasize the need for a holistic approach and broad perspective on the long-term evolution of the labor market to understand the causes of deflation. Moreover, the study of the microeconomic factors affecting the demand and supply of labor can be enhanced by properly framing individual behavior and choices within the structural transformations that have modified the supply of labor and the composition of the workforce.

In terms of policy implications, our results underline the importance of structural changes in the labor market in order to reduce the risk of deflation. The Japanese case shows that the reforms aimed to make the job market more flexible, and the progressive de-unionization observed in virtually all major economies can contribute to disentangling wage inflation from employment dynamics, weakening their monetary authorities’ capacity to achieve their inflation targets, thereby rendering the macroeconomy susceptible to deflation. More specifically, the precarization of the labor force weakens workers’ bargaining power on the one hand and attracts labor force workers with higher wage elasticity on the other, making it possible for firms to adjust on the extensive margin without a relevant increase in costs.

## Concluding remarks

This paper presents a parsimonious model of the labor market, in which the labor force is composed of primary and secondary workers. A wage Phillips curve, which includes the structural parameters of the labor market, is derived analytically. We find that the slope of the wage Phillips curve depends on the joint effects of the composition of the workforce, bargaining power of primary workers, and wage elasticity to supply of secondary workers.

The solution of the model allows for a qualitative assessment of the role of a series of structural changes observed in the Japanese economy in the flattening of the wage Phillips curve. None of these effects can generate the shifts in the wage Phillips curve, as observed in the data, in isolation. Our analysis suggests that the partial disentanglement of the dynamics of economic activity and wages is the result of deep modifications in the labor market and its long-term structural evolution. Therefore, any quantitative analysis must consider a long-term perspective and these structural factors.

In this study, we limit our analysis to the labor market in order to better highlight the causal nexus between its long-term evolution and the flattening of the wage Phillips curve. Future extensions could explicitly model the feedback effects of the sluggish dynamics of wages and aggregate demand.

## Supplementary Information

ESM 1 (PDF 202 KB)

## Data Availability

Data are publicly available at Ministry of Health Labor and Welfare (2020) and Statistics Bureau of (2021).

## Footnotes

1 See for example Chen (2018) and Fukao and Perugini (2018), and Stansbury and Summers (2020) about the decline in the bargaining power of workers; and Bank of Japan (2018) on the elasticity of the supply of labor to wages in the secondary market; Gordon (2017) and Kawaguchi and Ueno (2013) on the composition of the workforce. Del Negro Del Negro et al. (2020) propose a structual vectore autoregression (SVAR) analysis that includes different measures of employment and wage dynamics but abstracts from the duality in the labor market.

2 More precisely, following Statistics Bureau (2021), secondary workers include: part-time workers, dispatched workers from labor agencies,, contract/entrusted employees, and a generic “other” category.

3 Japanese firms rarely lay off their primary workers. In 2020, for example, in the mid of COVID–19 recession, real Gross Domestic Product (GDP) fell by unprecedented 28.1%, and yet, the unemployment rate rose only slightly to 2.8%.

4 The details of this calculation are provided in the Supplementary Note.

5 The decline of trade union density in Japan is in line with the global trend. According to the OECD data, the percentage of unionized members in Japan went from 31*%* in 1980 to 16.8*%* in 2021, whereas the average value of the OECD declined from 36.5*%* to 15.8*%* in the same period.

## Code Availability

Codes are available upon request.
