# Supplementary material for: Dual labor market and the “Phillips curve puzzle”: the Japanese experience
Source: J Evol Econ. 2022 Aug 5;32(5):1419–35. doi: 10.1007/s00191-022-00781-8 (PMC9362386; doi:10.1007/s00191-022-00781-8)
Supplement: Supplementary file 1 — (PDF 202 KB) [file 191_2022_781_MOESM1_ESM.pdf]

Dual Labor Market and the "Phillips Curve Puzzle": The Japanese Experience

Supplementary Note

Hideaki Aoyama, Corrado Di Guilmi

Yoshi Fujiwara, Hiroshi Yoshikawa

Abstract

This supplementary note presents the details for the derivation of the solution of the model and a basic explanation of the concept of *dimensionality*, which plays essential role in solving the model. Equations that appear also in the main text have the same numeration as in the paper.

1 Solving the model

Toward the goal of solving the model, we first rewrite Eq. (8) as follows:

$$\frac{w_2}{\alpha\, c\, A} = \left(L_1 + c\, B\, w_2^{\beta}\right)^{-(1-\alpha)}. \tag{S.1}$$

Let us introduce the scaled variable v and g:

$$v \equiv \frac{L_1^{\beta(1-\alpha)}}{(\alpha\, c\, A)^{\beta}} w_2^{\beta}; \quad g \equiv c\, B\, (\alpha\, c\, A)^{\beta}\, L_1^{-1-\beta(1-\alpha)}. \tag{16}$$

which we can use to rewrite Eq.(S.1) as follows:

$$v = (1 + g\, v)^{-\beta(1-\alpha)}, \tag{S.2}$$

Since solving the model amounts to finding the equilibrium w_2 which is equivalent to v, we focus on Eq.(S.2). Both v and g are dimensionless[1] quantities

[1] This concept is explained in section 2.

in Eq.(S.2), making the following analysis straightforward. Using v, we can express L and W as follows:

$$L = L_1 \left[1 + \frac{g}{c} v \right], \tag{15}$$

$$W = \frac{g^{1/\beta} L_1^{1+1/\beta}}{\alpha c (cB)^{1/\beta}} \left[\gamma \, v^{-\alpha/(\beta(1-\alpha))} + (1-\gamma)\, \alpha \, g \, v^{1+1/\beta} \right]. \tag{S.3}$$

Accordingly, the average wage is given by:

$$\bar{w} = \frac{W}{L} = \left(\frac{L_1}{B} \right)^{1/\beta} Z(\alpha, c, \beta, \gamma, g, v). \tag{S.4}$$

The coefficient $(L_1/B)^{1/\beta}$ is the *only* factor with the same dimension as $\bar{w}$. The function $Z(\alpha, c, \beta, \gamma, g, v)$ is the following dimensionless function of the dimensionless parameters $\alpha, c, \beta, \gamma, g$ and $v = v(g)$:

$$Z(\alpha, c, \beta, \gamma, g, v) = \frac{g^{1/\beta}}{\alpha \, c^{1+1/\beta}} \left[\gamma \, v^{-\alpha/(\beta(1-\alpha))} + (1-\gamma)\, \alpha \, g \, v^{1+1/\beta} \right] \Big/ \left[1 + \frac{g}{c} v \right]. \tag{S.5}$$

The Phillips curve, which we express as a relationship between the average wage and employment, $\bar{w}(L)$, is obtained by eliminating g (and $v = v(g)$) from Eq. (15) and Eq. (S.4).

Now, the second term in parentheses on the right-hand-side of Eq.(S.2), gv is the ratio cL_2/L_1. Therefore, if L_1 and L_2 measured in efficiency units are different in order, we can approximate it around the larger term. In other words, if $L_1 \gg cL_2$, namely, if the primary workers dominate in efficiency in production, we expand the right hand side around for $gv \ll 1$ or equivalently $g \ll 1$ (note that large L_1 implies small g because of Eq.(**??**)), or, if otherwise, we use $g \ll 1$ expansion.

This analytical result is valid for $g \ll 1$, or

$$L_1 \ll \left(B A^\beta \right)^{1/(1+\beta(1-\alpha))}, \tag{S.6}$$

where we ignored irrelevant quantities of order one, namely c and α.

The small-g perturbative solution of Eq.(S.2) is the following:

$$v = 1 - \sigma g + \frac{1}{2}\sigma(1+3\sigma) g^2 - \frac{1}{3}\sigma(1+2\sigma)(1+4\sigma) g^3 + \cdots, \tag{S.7}$$

where $\sigma \equiv \beta(1-\alpha)$. By substituting Eq.(17) into Eqs.(15), (S.4), and (S.5), we obtain the following equations:

$$L = L_1 \left[1 + \frac{g}{c} - \sigma \frac{g^2}{c} + \cdots \right] \tag{S.8}$$

$$\bar{w} = \left(\frac{L_1}{B} \right)^{1/\beta} \frac{g^{1/\beta}}{\alpha \, c^{1+1/\beta}} \left[\gamma + \left(\alpha - \frac{\gamma}{c} \right) g + \cdots \right]. \tag{S.9}$$

In order to eliminate g from these two equations and obtain functional relationship between L and $\bar{w}$, we first solve Eq.(S.7) for small g:

$$g = c\left(\frac{L}{L_1} - 1\right) + c^2\sigma\left(\frac{L}{L_1} - 1\right)^2 + \frac{1}{2}c^3\sigma(\sigma-1)\left(\frac{L}{L_1} - 1\right)^3 + \cdots . \quad \text{(S.10)}$$

Note that this is a perturbative series in $L/L_1 - 1 = L_2/L_1 \ll 1$. By substituting this expression into Eq.(S.8), we obtain the following expression of $\bar{w}$:

$$\bar{w} = \frac{\gamma}{\alpha\, c}\left(\frac{L_1}{B}\right)^{1/\beta}\left(\frac{L}{L_1} - 1\right)^{1/\beta}\left[1 + \frac{c(\alpha+\gamma-\alpha\gamma)-\gamma}{\gamma}\left(\frac{L}{L_1} - 1\right) + \cdots\right]. \quad (18)$$

Thus, the average wage $\bar{w}$ is a monotonically increasing function of total employment, L. In particular, the Phillips curve has the expected sign of slope: it is downward sloping on the wage-unemployment plane. We find from the leading term of this expression that the slope of the curve depends on two key factors, $\gamma/(c\,\alpha B^{1/\beta})$ and $1/\beta$.

The above analysis assumes $L_1 \gg cL_2$. We can make similar analysis in the case of $L_1 \ll cL_2$, which is not developed here as it is not relevant for the analysis presented in the paper.

2 Dimensional Analysis

In this section, we discuss the dimensions of various parameters and variables in our model, as this concept of dimensionality helps to solve the Model, as is explained in the first half of this note.

Dimensions play important role in various fields of natural science. Basic dimensions in natural science are: Length, Weight, Time and Charge. In any equation that deals with natural quantities, the dimension of the left-hand side has to be equal to the dimension on the right-hand side. For example, "1 [in meter] = 1 [in kilogram]" does not make sense. For this reason, we often learn a lot by simply looking at the dimensions of the constants and variables. This is called "dimensional analysis".

Also, dimensionless quantities play important roles in analysis: The most famous dimensionless constant is the fine structure constant $\alpha = e^2/\hbar c = 1/137.035...$ (in cgs units), where e is the unit of electric charge, $\hbar$ is the reduced Planck's constant and c is the speed of light. As this quantity is dimensionless, α has this value, regardless of whether length is measured in meters or feet, or whether weight is measured in kilogram or pounds, and so on.

Our analysis of the model benefits greatly by the dimensional analysis. Let us examine dimensional properties of quantities in our model. We denote the dimension of the number of workers by $\mathbf{H}$, unit of value, like the dollar or yen, by $\mathbf{V}$, and time by $\mathbf{T}$.

First, the parameters c, α, β and γ are dimensionless by their definitions.

Dimensions of the fundamental variables are the following:

$$\dim Y = \mathbf{V}\,\mathbf{T}^{-1}, \tag{S.11}$$

$$\dim L_{1,2} = \mathbf{H}, \tag{S.12}$$

$$\dim w_{1,2} = \mathbf{H}^{-1}\,\mathbf{V}\,\mathbf{T}^{-1}, \tag{S.13}$$

as Y is value created per a unit of time (yen per year, for example), $L_{1,2}$ are number of workers, and $w_{1,2}$ are value per person per time. From these, we find the following dimensions of the parameters:

$$\dim A = \mathbf{H}^{-\alpha}\,\mathbf{V}\,\mathbf{T}^{-1}, \tag{S.14}$$

$$\dim B = \mathbf{H}^{1+\beta}\,\mathbf{V}^{-\beta}\,\mathbf{T}^{\beta}. \tag{S.15}$$

The former is obtained by the requirement that dimensions of the right-hand side and the left-hand side of Eq.(2) matches, and the latter similarly from Eq.(7). From these, we find that the scaled variable v (Eq.(16)) and the parameter g (Eq.(**??**)) are dimensionless. For this reason, the nonlinear equation Eq.(8), which plays a central role in our model but is rather complicated, is simplified to a form much simpler and easier to analyse, Eq.(S.2).
